# Integrating clinical trial landscapes and bibliometric analysis: unveiling the impact of PD-1/PD-L1 inhibitors on renal cell carcinoma research and therapeutic trajectories summary

**DOI:** 10.3389/fimmu.2025.1578838

**Published:** 2025-07-23

**Authors:** Yuanbin Huang, Xinmiao Ma, Hengxing Zhu, Chen Shen, Ke Hu, Yang Yu, Aoyu Yang, Zhuo Liu, Chuanyang Liu, Wenrui Shi, Wei Wang, Xueyan Xia, Jiawen Wang, Xiancheng Li

**Affiliations:** ^1^ Department of Urology, Second Affiliated Hospital of Dalian Medical University, Dalian, Liaoning, China; ^2^ Division of Pancreatic Surgery, Department of General Surgery, West China Hospital, Sichuan University, Chengdu, Sichuan, China; ^3^ Department of Obstetrics and Gynecology, Shengjing Hospital of China Medical University, Shenyang, Liaoning, China; ^4^ Department of General Medicine, Liaoning Cancer Hospital of Dalian University of Technology, Shenyang, Liaoning, China; ^5^ Medical Literature Retrieval Department, Dalian Medical University, Dalian, Liaoning, China; ^6^ Department of Urology, Shengli Clinical Medical College of Fujian Medical University, Fujian Provincial Hospital, Fuzhou University Affiliated Provincial Hospital, Fuzhou, Fujian, China

**Keywords:** renal cell carcinoma, PD-1/PD-L1, immunotherapy, bibliometric analysis, research trends

## Abstract

**Background:**

Renal cell carcinoma (RCC) is a prevalent tumor of the urinary system. Beyond surgical treatment, targeted therapies and immunotherapies are the primary therapeutic options for RCC. Although immunotherapy has been extensively studied, research on the association between the immune checkpoint PD-1/PD-L1 and RCC remains relatively novel. Thus, we aim to assess the global scientific outcomes of studies focusing on PD-1/PD-L1 in RCC from 2005 to 2024 and to identify emerging research trends.

**Methods:**

Data were collected from the Web of Science Core Collection using a predefined search strategy. A total of 1,597 articles were ultimately included. In addition, 258 clinical trials registered on ClinicalTrials.gov from 2011 to 2024 were reviewed to evaluate the translational progress and global research activity. The articles were visualized and analyzed using GraphPad Prism and the bibliometric tools CiteSpace and VOSviewer.

**Results:**

The number of publications in this field has shown a consistent upward trend, with a marked increase starting in 2013 and peaking in 2021. At the national level, the United States ranks first in both the number of publications (n = 625) and total citations (n = 68,687). At the institutional level, Harvard University is the most productive and most cited institution among all contributors. The Journal for Immunotherapy of Cancer published the highest number of articles (n = 66), whereas the New England Journal of Medicine was the most frequently co-cited journal (n = 1,300), indicating its authoritative influence. Notable individual contributors, including Choueiri TK and Motzer RJ, have played pivotal roles in advancing research, particularly in first-line combination therapies for RCC. Frequently occurring keywords such as “immunotherapy,” “nivolumab,” “expression,” and “immune checkpoint” reflect current research hotspots and suggest future directions in this domain. Clinical trial analysis revealed that most studies were early-phase, sponsor-driven, and regionally heterogeneous in design and outcomes, highlighting both the promise and the ongoing challenges of clinical translation.

**Conclusion:**

This study provides domestic and international researchers with a comprehensive overview of the current research landscape surrounding PD-1/PD-L1-based immunotherapy in RCC. Moreover, it identifies emerging research trends and translational progress, thereby offering valuable guidance for subsequent scientific investigations and clinical application.

## 1 Introduction

Renal cell carcinoma (RCC) is among the most common malignancies of the urinary system, with its incidence steadily increasing due to aging populations, obesity, and environmental factors (1, 2). RCC encompasses several histological subtypes, of which clear cell RCC (ccRCC) is the most prevalent, accounting for approximately 70%–80% of cases (3). Other subtypes include papillary RCC and chromophobe RCC. RCC is often asymptomatic in its early stages, resulting in late-stage diagnosis and poor prognosis (4). Approximately 25% of RCC patients present with metastasis at diagnosis, and the 5-year survival rate in metastatic RCC remains below 10% (4, 5). Traditional therapeutic strategies for advanced RCC, including surgical resection and targeted therapies such as vascular endothelial growth factor (VEGF)-targeted tyrosine kinase inhibitors (TKIs) and immune checkpoint inhibitors (ICIs), have improved clinical outcomes to some extent (**Supplementary Table 1**) (6, 7). However, issues like acquired drug resistance, immune escape, and adverse events remain major limitations (8, 9). RCC is considered a highly immunogenic tumor, yet its progression is closely associated with immune dysregulation (10). This includes T cell exhaustion, impaired antigen presentation, and the expansion of immunosuppressive cell populations such as myeloid-derived suppressor cells (MDSCs) and regulatory T cells (Tregs) (11–13). In addition, dysregulation of apoptosis pathways also contributes to immune escape by enabling tumor cells to resist immune-mediated cytotoxicity and evade elimination by cytotoxic T lymphocytes and natural killer (NK) cells (14). Immune checkpoint molecules—such as programmed cell death protein 1 (PD-1), its ligand PD-L1, cytotoxic T-lymphocyte-associated antigen 4 (CTLA-4), and T-cell immunoglobulin and mucin-domain containing-3 (TIM-3)—play pivotal roles in suppressing anti-tumor immunity (15). Among these, the PD-1/PD-L1 axis is particularly critical in the immune evasion of RCC. PD-1 is an inhibitory receptor expressed on activated T cells, while PD-L1 is frequently overexpressed on RCC tumor cells and antigen-presenting cells within the tumor microenvironment (16). Their interaction results in T cell exhaustion, impaired cytokine production, and reduced cytotoxic function, collectively contributing to tumor immune escape (17). Notably, RCC exhibits a highly immunosuppressive tumor microenvironment with elevated PD-L1 expression, which correlates with poor prognosis and aggressive tumor phenotypes (18). As a result, blockade of the PD-1/PD-L1 pathway using ICIs has emerged as a promising therapeutic approach in RCC. ICIs are monoclonal antibodies that restore antitumor immunity by blocking inhibitory checkpoint pathways such as PD-1/PD-L1, thereby reversing T cell exhaustion and enhancing cytotoxic activity (19). In recent years, ICIs—particularly those targeting PD-1 or PD-L1—have demonstrated significant survival benefits in advanced RCC, as shown in several pivotal clinical trials, including CheckMate 025, CheckMate 214, and KEYNOTE-426 (20–22). Given these encouraging outcomes, understanding the mechanistic relevance of the PD-1/PD-L1 axis is essential not only for interpreting clinical responses but also for guiding the rational design of next-generation immunotherapies.

The advent of immunotherapy, particularly anti-PD-1/PD-L1 antibodies, has significantly improved overall survival (OS) in patients with advanced RCC (23). These agents counteract immune evasion by restoring T-cell function through blockade of the PD-1/PD-L1 axis, enabling durable tumor control. To translate these benefits into clinical decision-making, the International Metastatic RCC Database Consortium (IMDC) risk stratification system remains widely used for guiding treatment selection (24). Current systemic therapy has shifted toward combination strategies that integrate ICIs with targeted therapies or dual immunotherapy approaches, tailored to patients’ risk profiles (25, 26). These advances highlight the importance of comprehensive bibliometric and clinical trial analyses to understand evolving research trends and guide future therapeutic development.

Although PD-1/PD-L1 inhibitors have demonstrated significant clinical efficacy in the treatment of RCC, the research landscape surrounding these immune checkpoint targets has not yet been systematically mapped using bibliometric approaches. Previous studies have primarily focused on overarching trends in cancer immunotherapy or individual checkpoint molecules, often lacking integration with clinical trial data and failing to provide disease-specific insights. Therefore, a comprehensive and integrative evaluation is warranted to better elucidate the interplay between academic output and clinical translation in this field.

In this study, we aim to comprehensively characterize the research trajectory of PD-1/PD-L1 in RCC by integrating bibliometric data from 2005 to 2024 and over a decade of global clinical trial records. Specifically, we identify and analyze the top ten high-impact publications, influential authors and institutions, emerging research hotspots, and clinical validation efforts. Our analysis provides evidence-based insights to support future research prioritization, clinical trial design, biomarker development, and precision immunotherapy strategies in RCC.

## 2 Methods

### 2.1 Data sources and search strategy

We conducted a comprehensive literature search in the Web of Science Core Collection (WoSCC) on December 30, 2024. The search was limited to English-language publications between January 1, 2005 and December 30, 2024. The search formula was:

TS=(“PD-1” OR “PD1” OR “CD279” OR “programmed death 1” OR “PD-L1” OR “PDL1” OR “CD274” OR “B7-H1” OR “programmed death ligand 1”) AND TS=(“renal cancer” OR “renal cell carcinoma” OR “renal cell cancer” OR “RCC” OR “kidney cancer” OR “kidney cell carcinoma” OR “kidney cell cancer”). Here, “TS” indicates a topic search including titles, abstracts, author keywords, and Keywords Plus.

### 2.2 Study selection and data extraction

All retrieved bibliographic records are managed and de-duplicated. After removing duplicates, two authors (YBH and XMM) independently screened the titles and abstracts. Full texts were then assessed for eligibility by the same two reviewers.

The inclusion criteria were: (1) original research articles focusing on PD-1/PD-L1 in RCC; (2) English-language publications; (3) studies containing accessible bibliometric metadata (e.g., title, authors, affiliations, abstract, keywords, citations).

Exclusion criteria included: (1) non-scholarly publications, such as commentaries, editorials, letters to the editor, and conference abstracts; (2) document types including retracted publications, early access articles, book chapters, proceedings papers, or publications with an expression of concern; (3) duplicate publications or literature that cannot be fully obtained.

**Figure 1:**
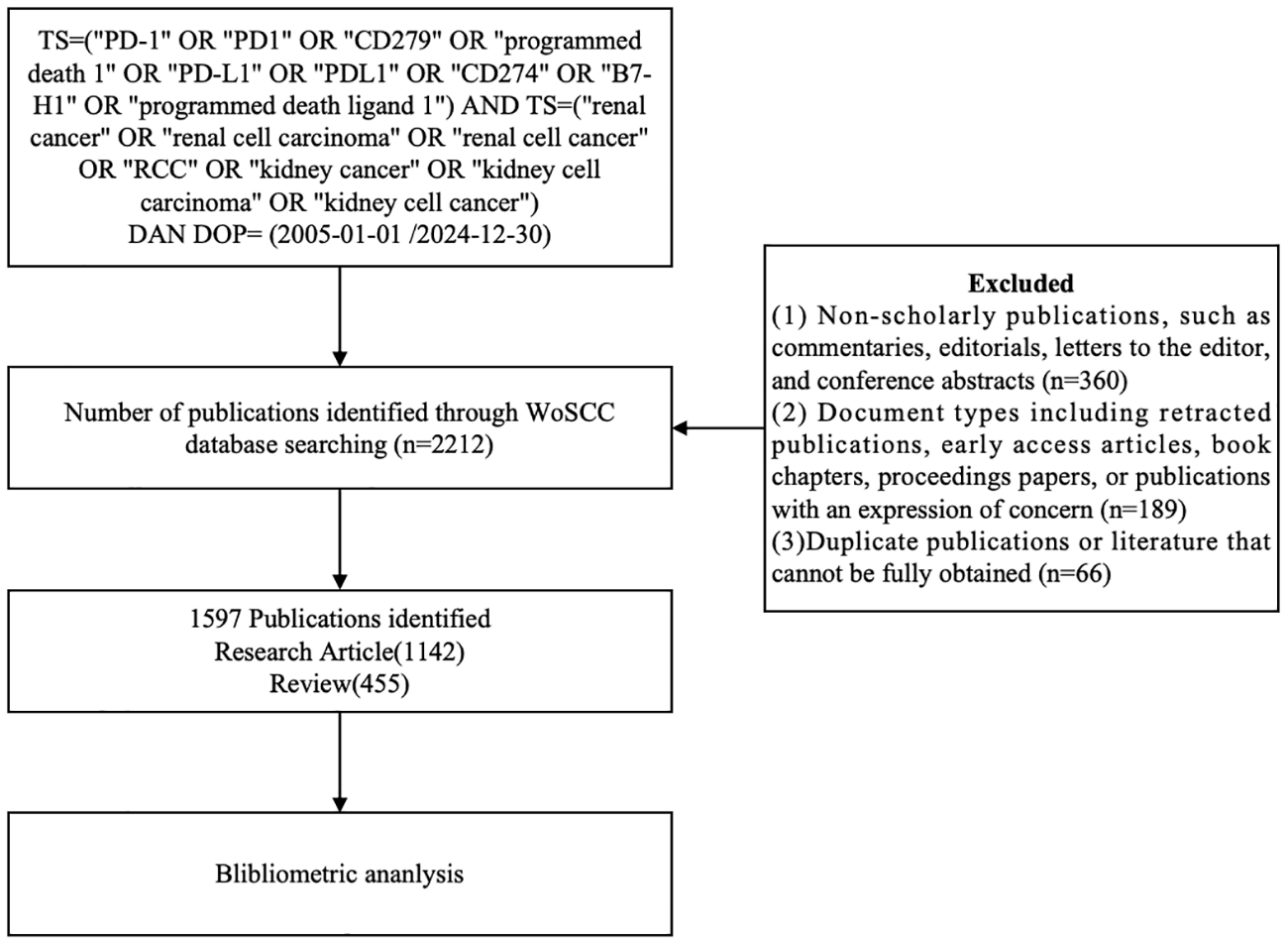
Flowchart illustrating the literature selection process, from database retrieval to final inclusion of articles (n=1597).

Discrepancies in study inclusion were resolved through discussion with a third reviewer (JWW). Inter-rater reliability for full-text screening was assessed using Cohen’s kappa statistic (κ = 0.84), indicating strong agreement between reviewers. A total of 1,597 publications met the inclusion criteria. A flow diagram (**Figure 1**) was used to depict the detailed selection process and ensure methodological transparency and reproducibility.

### 2.3 Bibliometric tools and parameters

All metadata (title, author, institution, journal, keywords, abstract, and cited references) were obtained from the WoSCC and exported in plain text format. GraphPad Prism (v8.0.2) was used to visualize annual publication trends and national contributions. Bibliometric analysis was conducted using VOSviewer (v1.6.18) and CiteSpace (v6.2.R4).

In VOSviewer, fractional counting was applied. The following thresholds were used: keywords (≥13 co-occurrences), authors (≥3 publications), countries (≥3 documents), and references (≥20 citations). Co-authorship, co-citation, and keyword clustering networks were generated and manually validated for interpretability (27).

In CiteSpace, time slicing was set from 2005 to 2024, with one-year intervals. Term sources included title, abstract, and author keywords. Node types were set to keyword, reference, author, and journal. Pathfinder and merged network pruning methods were applied. Citation bursts were detected using Kleinberg’s algorithm with a minimum burst duration of 2 years and a burst strength threshold of 3.5 (28).

All visualizations were cross-validated by two authors independently. Inter-rater agreement was assessed using Cohen’s kappa coefficient (κ = 0.85). Disagreements were resolved through discussion. No third reviewer was needed due to high agreement.

### 2.4 Clinical trial retrieval

Clinical trials were retrieved from ClinicalTrials.gov on December 31, 2024, using the following search terms: “renal cell carcinoma” AND (“PD-1” OR “PD-L1”) AND “immunotherapy”. Filters applied included: study type (interventional and observational), study status (all), and age group (adults, older adults and child). No restrictions were placed on study phase, location, or funding. Although no time filters were set, the earliest eligible trial included in our dataset was registered in 2011.

Studies were categorized as interventional or observational according to the classification on ClinicalTrials.gov. Trials were included only if they explicitly evaluated PD-1/PD-L1-based immunotherapy in RCC. The following variables were extracted: trial ID, title, intervention(s), study phase, status, sponsor, population, duration, and results. Positive outcomes (“YES”) were defined as trials that met their primary endpoints and reported clinical efficacy. All data were independently extracted by two authors and cross-verified.

## 3 Results

### 3.1 Analysis of annual publication trends

From 2005 to 2024, a total of 1597 publications related to PD-1/PD-L1 in RCC were retrieved from the WoSCC database, including 1142 research articles and 455 reviews. The annual publication output showed a continuous upward trend. Before 2012, the number of publications was relatively low (fewer than 10 per year), but a rapid increase was observed thereafter. This growth coincided with the accelerated development of ICIs and the approval of nivolumab—the first PD-1 inhibitor for RCC-by the FDA in 2015 (20). The publication count peaked in 2021, reaching 250 papers, likely due to the convergence of multiple factors, including the global expansion of cancer immunotherapy, increased clinical trial activity, and an initial boost in biomedical research funding during the early phase of the COVID-19 pandemic.

**Figure 2:**
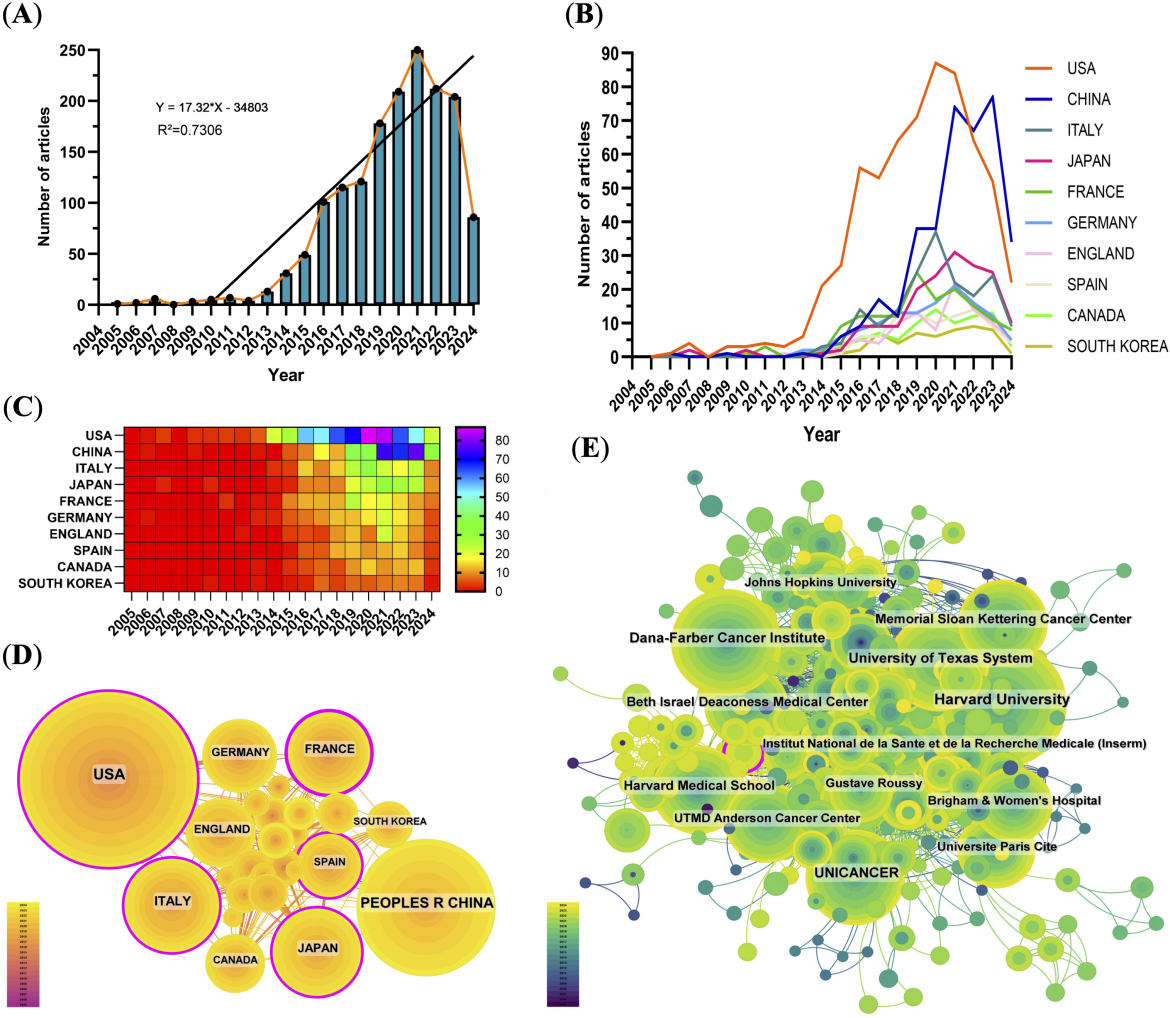
Global publication trends and collaboration analysis. **(A)** Annual number of publications from 2005–2024, peaking in 2021. **(B)** Line graph of annual publications by top contributing countries. **(C)** Heat map of publications by country, emphasizing major contributors. **(D)** International collaboration network; node size indicates publication volume, lines represent collaborative relationships, and purple outlines indicate high betweenness centrality. **(E)** Institutional collaboration network showing active connections among leading research institutions.

Analysis of national trends revealed that the United States initiated research in this area earlier and maintained leadership throughout most of the study period. In contrast, China exhibited a sharp increase in output after 2016. Its share of annual publications rose from under 10% before 2015 to nearly 30% in 2021. However, this surge was accompanied by a relatively lower average citation rate. Interestingly, some developed countries such as the United States and Italy showed a slight decline in publication numbers after 2021, possibly reflecting research disruptions and funding reallocation caused by the prolonged impact of the COVID-19 pandemic (**Figures 2A, B**).

### 3.2 Analysis of countries and institutions

Over the past two decades, the United States and China have emerged as the top two contributors to PD-1/PD-L1 research output. The United States ranked first with 625 publications (39.14%), followed by China with 375 publications (23.48%) (**Figure 2C**, **Table 1**). The U.S.’s leadership reflects its long-standing research infrastructure, stable funding, and global academic influence. In contrast, China’s rapid growth in publication volume underscores its recent strategic investments in biomedical research.

**Table 1:** Country-Level distribution of publications related to PD-1/PD-L1 inhibitor research in renal cell carcinoma.

Rank	Country/region	Article counts	Centrality	Percentage (%)	Citation	Citation per publication
1	USA	625	0.19	39.14%	68687	109.90
2	China	375	0	23.48%	11514	30.70
3	Italy	179	0.11	11.21%	14347	80.15
4	Japan	171	0.11	10.71%	14955	87.46
5	France	146	0.32	9.14%	19893	136.25
6	Germany	125	0.07	7.83%	15270	122.16
7	England	96	0.09	6.01%	15869	165.30
8	Spain	87	0.11	5.45%	9664	111.08
9	Canada	83	0.03	5.20%	11917	143.58
10	South Korea	55	0.01	3.44%	4360	79.27

However, quantitative output alone does not fully reflect academic impact. To account for potential time bias—where older publications naturally accumulate more citations—we normalized total citations by publication count to calculate citations per publication. The United States led not only in total citations (n = 68,687) but also in average citations per paper (109.90), indicating consistently high-impact research. China ranked second in total citations (n = 11,514) and seventh in citations per paper (30.70), revealing a noticeable gap between publication quantity and quality-adjusted impact. This discrepancy suggests differences in research visibility, influence, or maturity between the two countries.

International collaboration networks revealed that the United States formed strong cooperative ties with England, Germany, Italy, and Canada. In contrast, China’s collaborations were more regionally concentrated, primarily involving Japan, South Korea, and Spain (**Figure 2D**). Among the top 10 most productive institutions, eight were based in the United States and two in France (**Supplementary Table 2**). Harvard ranked first with 157 publications and 31,947 citations. Institutional collaborative networks showed that these leading institutions maintain dense collaborations, forming a highly interconnected research community (**Figure 2E**).

### 3.3 Analysis of journals and authors

**Figure 3:**
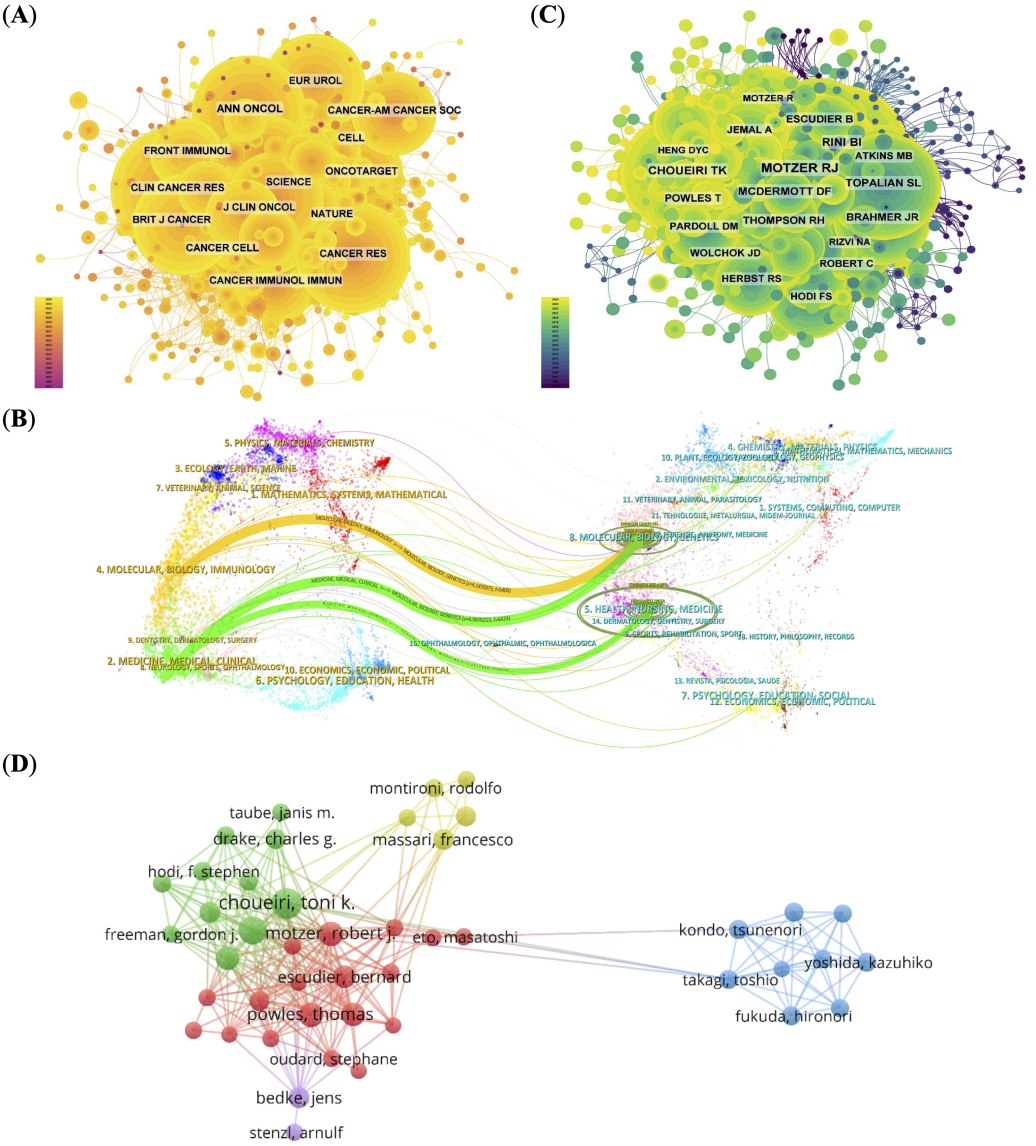
Co-citation and collaboration analyses for journals and authors. **(A)** Co-citation network of influential journals, with node size indicating citation frequency.**(B)** Dual-map overlay illustrating citation relationships between basic and clinical research domains. **(C)** Co-citation network of authors, identifying key contributors (e.g., Motzer RJ, Choueiri TK). **(D)** Author collaboration network, displaying research clusters and cooperation patterns.

The *Journal for Immunotherapy of Cancer* ranked first among the top 10 journals with 66 articles (4.13%) and the highest impact factor (IF) of 10.3 (**Supplementary Table 3**). The impact of journal is assessed by its co-citation frequency, reflecting its influence within the scientific community. The top 10 journals by co-citation count each exceeded 600 citations. The *New England Journal of Medicine* led with 1300 co-citations, and the co-citation network highlighted strong associations among leading journals (**Figure 3A**, **Supplementary Table 4**).

To further understand citation dynamics, we used a dual-map overlay of journals (**Figure 3B**). This visualization displays the citing journals on the left and cited journals on the right, with colored paths representing major citation trajectories. Two dominant citation paths were observed (1): from “Molecular, Biology, Immunology” to “Molecular, Biology, Genetics”, and (2) from “Medicine, Medical, Clinical” to both “Molecular, Biology, Genetics” and “Health, Nursing, Medicine”.

These patterns indicate a pronounced unidirectional flow of knowledge from basic sciences (e.g., molecular biology, immunology) to clinical fields, reflecting an active but asymmetric translational research model. While foundational discoveries are widely adopted in clinical oncology, reverse citations—from clinical practice back to basic science—are relatively sparse. This asymmetry suggests that despite growing interdisciplinary links, the field may still suffer from structural silos, with limited feedback mechanisms bridging clinical insights back to the laboratory. Strengthening this bidirectional integration could enhance the translational efficiency and innovation potential in PD-1/PD-L1-related RCC research.

Analyzing authors and their collaborative patterns reveals important insights into the structural dynamics and leadership of RCC immunotherapy research. The top 10 most prolific authors accounted for 306 papers (19.16%), with McDermott DF, and Motzer RJ leading the field. Notably, Motzer RJ (951 citations) and Choueiri TK (554 citations) received the highest number of co-citations, indicating not only research output but also sustained influence within the academic community (**Figure 3C**; **Supplementary Table 5**).

Over 80 authors received more than 50 co-citations, reflecting a well-established and impactful core group of investigators. These citation patterns suggest that RCC immunotherapy research is driven by a relatively concentrated network of experts with strong academic visibility. The author collaboration network (**Figure 3D**), visualized using VOSviewer, reveals five distinct clusters. The red and green clusters are tightly connected, with Choueiri TK and Motzer RJ at their core, reflecting long-standing and productive institutional collaborations that have helped shape therapeutic strategies in the field. In contrast, the blue cluster appears more insular, likely representing specialized research niches or institutions with focused but less externally integrated programs. This structural division may reflect differences in funding sources, institutional mandates, or regional research priorities.

### 3.4 Analysis of references

The co-citation network constructed using CiteSpace contains 1160 nodes and 5825 links, indicating high interconnection among the core literature in the field. The top 10 most co-cited articles (**Supplementary Table 6**) each received over 100 citations, with 7 of them published in the *New England Journal of Medicine*, 5 of which were led by Motzer RJ (20, 21, 29–31), highlighting his pivotal role in the clinical and foundational research of RCC immunotherapy.

**Figure 4:**
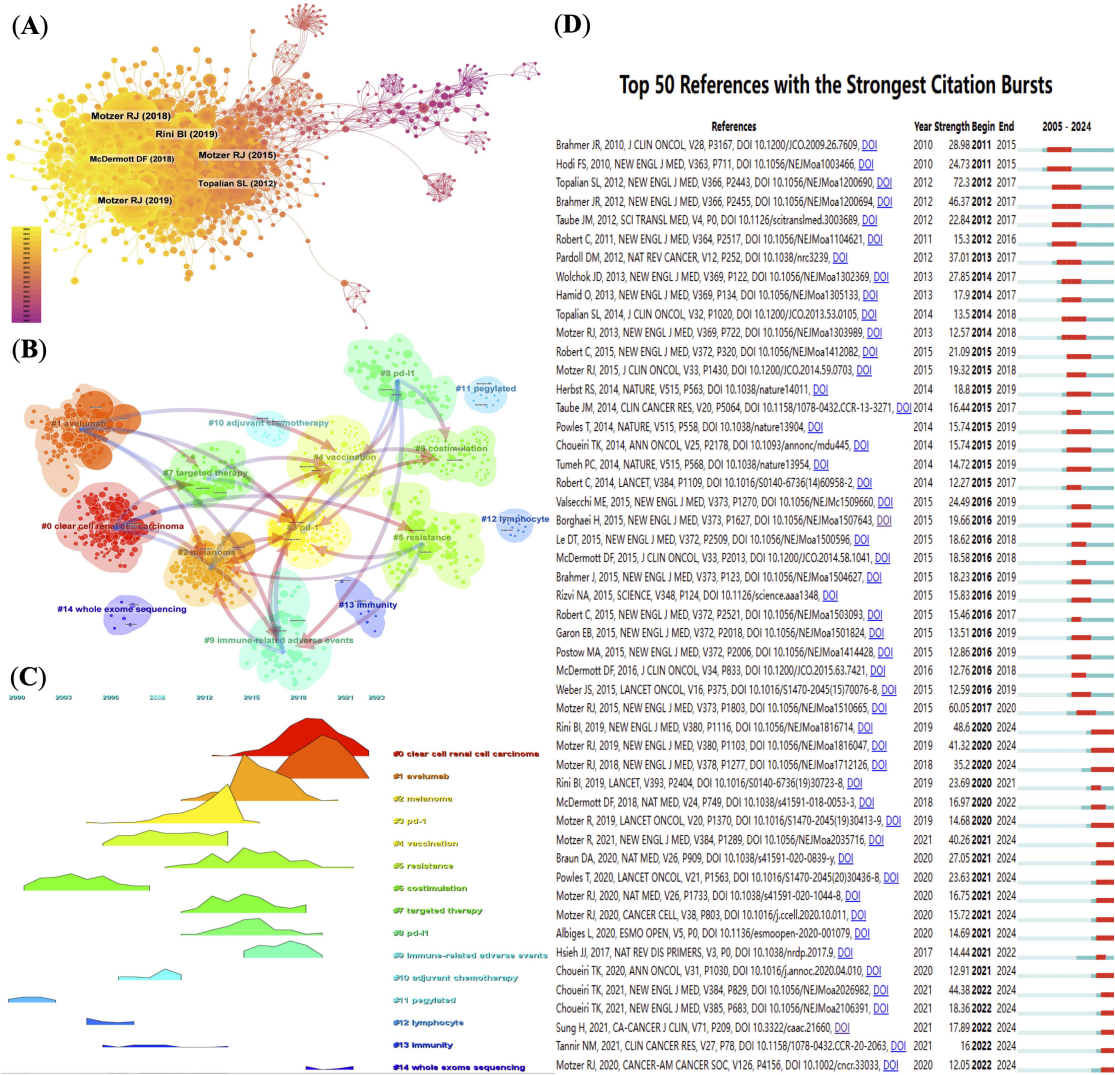
(Analysis of reference co-citations and thematic evolution. **(A)** Network map of co-cited references, node size proportional to citation count. **(B)** Clustering of co-cited literature into thematic groups. **(C)** Timeline visualization of thematic cluster citation peaks over time. **(D)** Top 50 references with strongest citation bursts, indicating pivotal studies.

From a temporal perspective, the evolution of co-cited clusters (**Figures 4B, C**) reveals that the research topics in this field have shifted from early studies centered on basic immunological mechanisms such as “costimulation,” “immunity,” and “lymphocytes,” to later-stage research focusing on clinical translational topics such as “immune checkpoint inhibition,” “combination therapy,” and “immune-related adverse events.” This shift reflects the deepening transition from basic research to therapeutic applications and toxicity management.

The burst citation analysis reveals certain studies that gained high attention within a short period. For example, Topalian et al.’s 2012 paper showed the highest burst intensity (72.3), marking a milestone in PD-1 research (32). In CiteSpace, “burst intensity” refers to the rate and magnitude of a paper’s citation frequency sharply increasing within a specific time window, reflecting the paper’s rapid rise to prominence in the academic community. This metric helps identify studies that have had a significant impact on the academic evolution of the field. A high burst intensity value suggests that the paper made a substantial contribution during that period, often associated with groundbreaking findings or developments. In recent years, burst literature has increasingly focused on predictive biomarkers, tumor immune microenvironment, and whole-exome sequencing, reflecting the growing focus on precision oncology. **Figure 4D** illustrates the co-citation frequency of representative gene mutations and immune markers related to RCC immunotherapy, visualizing current research hotspots and emerging trends.

### 3.5 Keywords analysis

**Figure 5:**
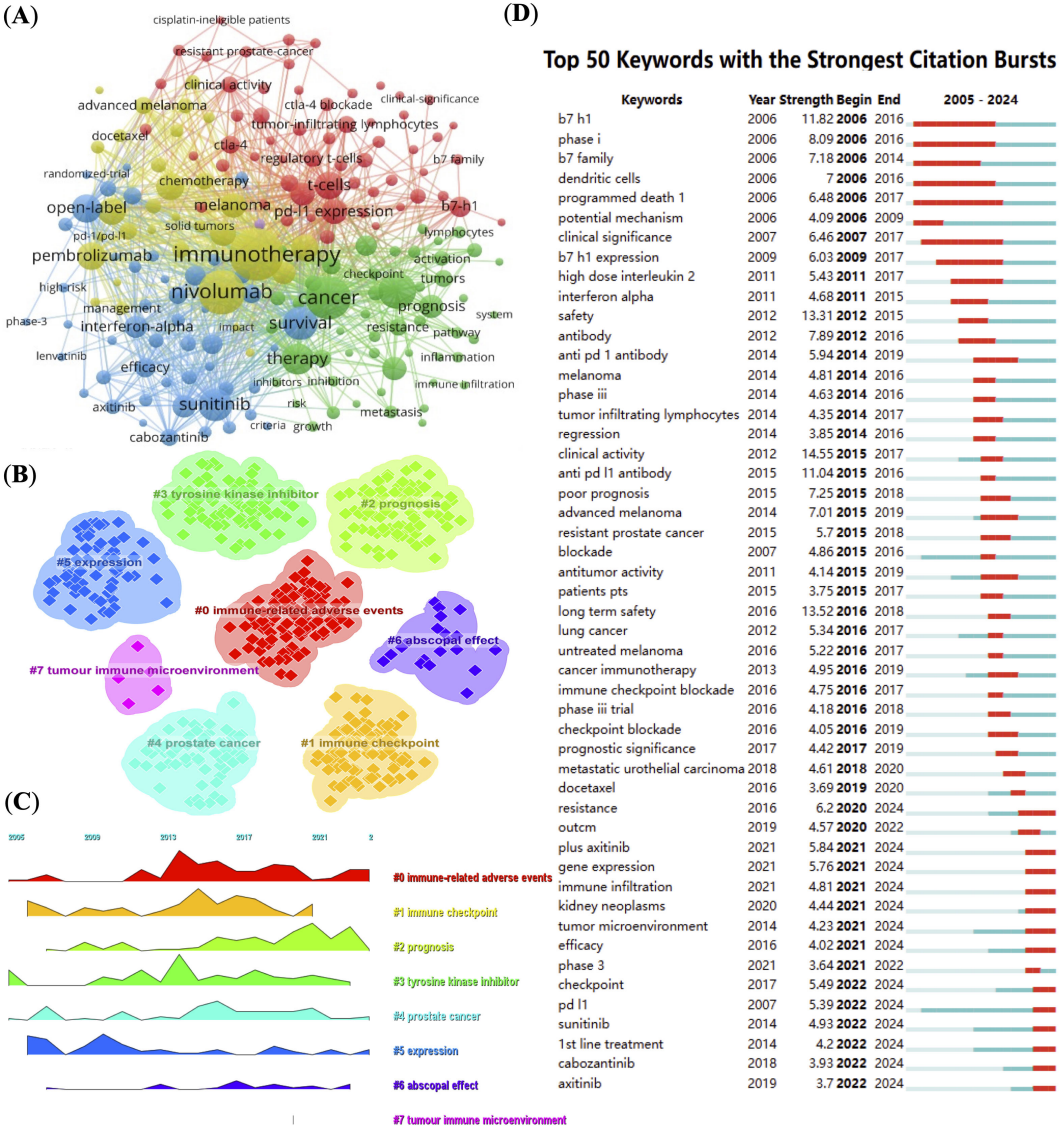
Keyword network and trend analysis. **(A)** High-frequency keyword co-occurrence network, node size indicating keyword frequency. **(B)** Clustering of keywords into main research themes. **(C)** Timeline visualization showing keyword prominence and temporal dynamics. **(D)** Top 50 keywords with strongest citation bursts, highlighting emerging research topics.

By analyzing the keywords, we can quickly understand the situation and development direction of a field. The most common keywords include “immunotherapy” (n = 553), “nivolumab” (n = 404), “cancer” (n = 376), “expression” (n = 268) and “survival” (n = 224) (**Table 2**). We constructed a network of 189 keywords, each occurring at least 13 times, after removing non-informative terms, resulting in five distinct clusters (**Figure 5A**). To filter non-informative terms, we employed a systematic methodology that involved the removal of common stopwords, such as “and,” “the,” “of,” and other frequently occurring but contextually irrelevant terms. Additionally, terms that appeared excessively without contributing specific meaning to the research focus, such as general technical terms or overly broad concepts, were also excluded. The remaining terms were carefully selected based on their frequency of occurrence (at least 13 times), ensuring that only keywords highly relevant to the research themes were retained.

**Table 2:** High frequency keyword table.

Rank	Keyword	Counts	Rank	Keyword	Counts
1	Immunotherapy	553	11	Ipilimumab	150
2	Nivolumab	404	12	Immune checkpoint inhibitors	146
3	Cancer	376	13	Blockade	141
4	Expression	268	14	PD-L1 expression	127
5	Survival	224	15	Safety	126
6	Sunitinib	215	16	Melanoma	124
7	Therapy	203	17	Prognosis	116
8	Open-label	164	18	PD-1 blockade	112
9	Pembrolizumab	160	19	Clear cell renal cell carcinoma	110
10	T-cells	155	20	Everolimus	108

We used CiteSpace (v6.2.R4) to map the evolution of keyword clusters and trends in RCC and PD-1/PD-L1 literature. **Figures 5B, C** offer a keyword clustering analysis that maps the evolution of research themes in RCC and PD-1/PD-L1 immunotherapy. Seven distinct clusters are identified, each corresponding to a different research focus. Cluster 0 emphasizes “immune-related adverse events,” which is crucial for understanding the safety and side effects of immune checkpoint inhibitors. Cluster 2 and 3 focus on “prognosis” and “tyrosine kinase inhibitors,” reflecting a growing interest in treatment outcomes and combination therapies. Cluster 5 centered on “expression,” represents foundational research into gene and protein expression in RCC. Cluster 7 highlights the “tumor immune microenvironment,” a key area in immunotherapy research. Other clusters, such as “immune checkpoint”, “prostate cancer”, and “abscopal effect”, further underscore the diverse research interests and the integration of immunotherapy across different cancer types. Keyword evolution mapping using CiteSpace highlighted shifts in research focus over time. Early literature emphasized basic concepts such as “expression” and “immune checkpoint,” while recent years showed an increased emphasis on “resistance,” “immune infiltration,” and “tumor microenvironment.” These transitions suggest a shift from molecular characterization to understanding clinical resistance mechanisms and therapeutic optimization. Keyword burst detection was conducted using CiteSpace’s built-in burst detection algorithm based on Kleinberg’s algorithm, which identifies keywords with a significant increase in frequency over a defined time period. The burst strength indicates the magnitude of this increase, and we set the default parameters of CiteSpace to determine citation bursts. Of the 354 most frequent keywords identified, we selected the top 50 with the strongest burst strength for analysis (**Figure 5D**). Earlier keywords like “expression” were dominant, often reflecting gene or protein expression profiles in specific disease contexts. Subsequently, keywords such as “tyrosine kinase inhibitors,” “immune checkpoints,” and “immune-related adverse events” gained prominence. In recent years, prognosis-related keywords have risen in importance. By 2024, frequently cited burst keywords included “resistance,” “gene expression,” “immune infiltration,” “tumor microenvironment,” “efficacy,” “checkpoint,” “PD-L1,” “sunitinib,” “1st line treatment,” “cabozantinib,” and “axitinib,” indicating current research frontiers in PD-1/PD-L1 immunotherapy for RCC.

### 3.6 Clinical trial data analysis

**Figure 6:**
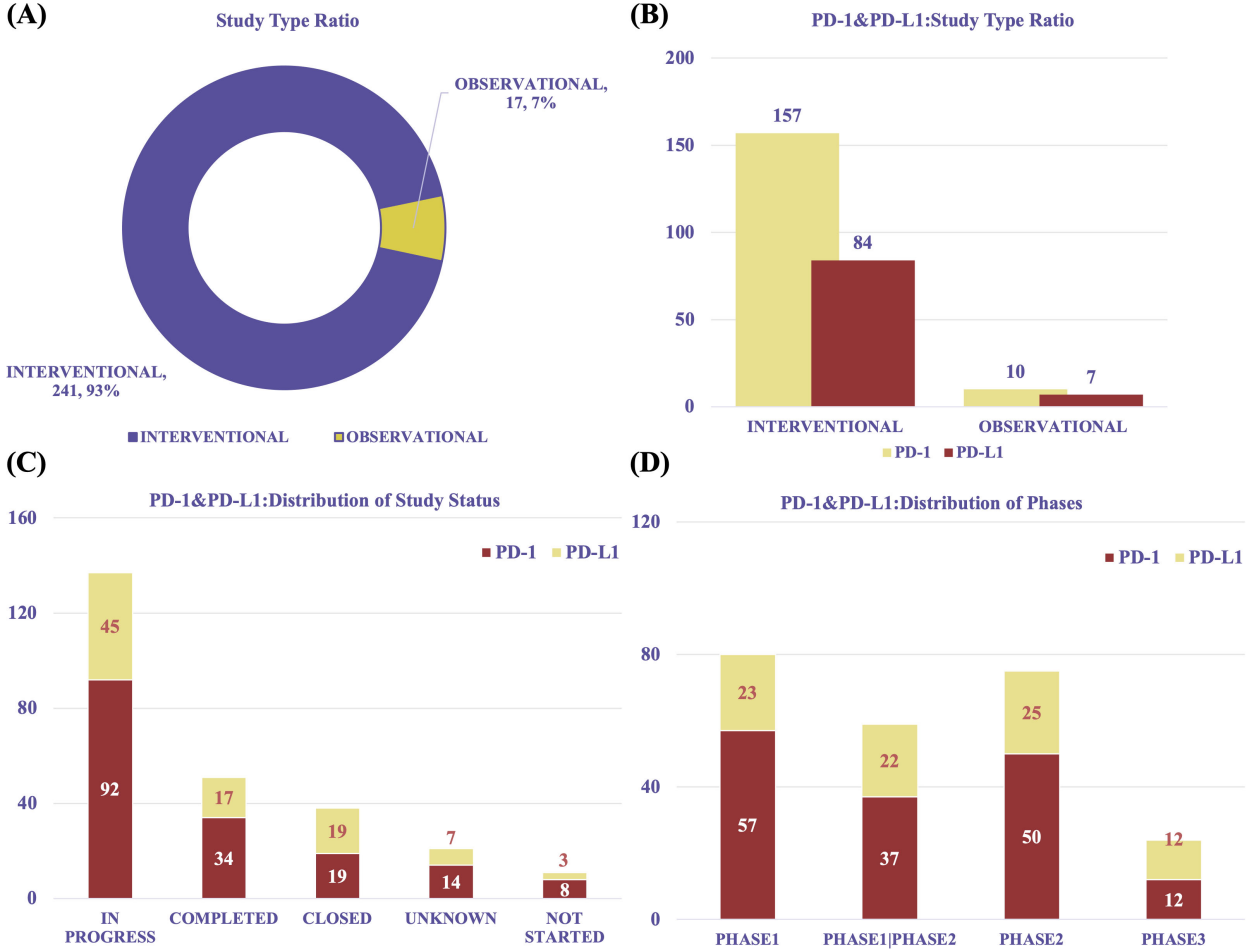
Overview of clinical trial characteristics. **(A)** Trial type distribution (interventional *vs* observational). **(B)** Proportion of PD-1 and PD-L1 trials by trial type. **(C)** Distribution of trial statuses (ongoing, completed, terminated). **(D)** Distribution of clinical trial phases, showing predominance of early-phase trials.

We analyzed data from 258 global clinical trials investigating PD-1 and PD-L1 therapies for RCC. Since 2015, research in this field has grown rapidly (**Supplementary Figure 1A**). The majority of studies were interventional (n = 241, 93%), while observational studies accounted for only 7% (n = 17). Among interventional trials, those targeting PD-1 (n = 157) outnumbered those focusing on PD-L1 (n = 84). Similarly, observational studies included 10 PD-1 trials and 7 PD-L1 trials (**Figures 6A, B**).

Most participants were adults or elderly (n = 251, 97%), with only 3% of trials involving pediatric populations. Gender information was frequently unspecified (**Supplementary Figures 1B, C**). Across multiple dimensions, PD-1–focused studies were consistently more prevalent than PD-L1–focused ones.

In terms of trial status, 137 studies were in progress, including 92 PD-1 and 45 PD-L1 trials. Additionally, 51 trials were completed (PD-1: n = 34; PD-L1: n = 17), and 38 were closed (PD-1: n = 19; PD-L1: n = 19) (**Figure 6C**). The majority of trials were early-phase studies, including Phase I (n = 80), Phase I/II (n = 59), and Phase II (n = 75), with relatively few advancing to Phase III (n = 24) (**Figure 6D**). This distribution reveals a significant translational gap in the clinical development of PD-1/PD-L1 therapies for RCC. Despite promising preclinical and early-phase results, progression to late-stage trials remains limited, possibly due to challenges in patient recruitment, long-term efficacy assessment, regulatory barriers, and financial constraints.

**Figure 7:**
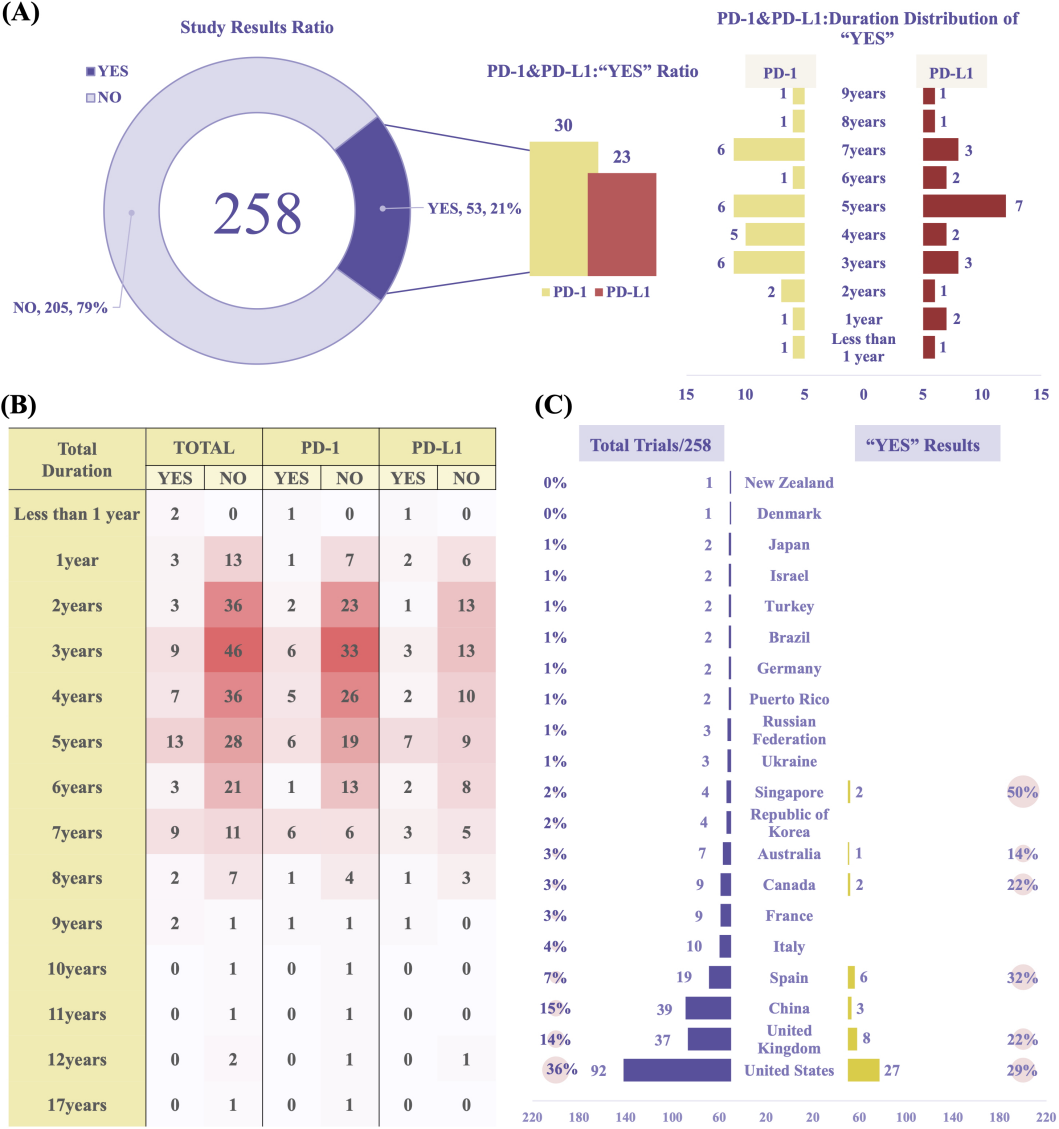
Clinical trial outcomes and geographical analysis. **(A)** Proportion of trials reporting positive outcomes (“YES” results). **(B)** Heatmap illustrating trial durations and outcomes (PD-1 *vs* PD-L1 studies). **(C)** Geographic distribution and “YES” outcome rates of PD-1/PD-L1 trials in RCC. Data reflect combined results from both PD-1 and PD-L1 studies.

The overall proportion of trials with positive results (“YES”) was 21%, with 53 studies meeting their pre-specified primary endpoints. To evaluate trial outcomes, we classified studies based on endpoint achievement and result availability. A “YES” result was defined as a study that met its primary endpoint according to pre-specified criteria and reported efficacy outcomes in peer-reviewed publications or trial result databases. A “NO” result included studies that were terminated prematurely, failed to meet their primary endpoint, or lacked publicly available results. Among the 53 “YES” studies, 30 involved PD-1 and 23 involved PD-L1. Notably, the number of studies exceeding 5 years in duration declined significantly (**Figure 7A**). The heatmap analysis, combined with study duration, revealed the distribution patterns of research activity, with most trials lasting between 2 to 5 years. PD-L1–related studies exhibited a more dispersed duration pattern compared to PD-1 studies. This 2–5-year timeframe likely reflects the typical period needed to evaluate short- to medium-term efficacy and safety endpoints in immuno-oncology, such as progression-free survival or objective response rate. The marked decline in studies exceeding 5 years may indicate challenges in sustaining long-term follow-up, including declining patient adherence, limited funding continuity, and pressure to report interim findings early. This pattern suggests that current RCC immunotherapy trials may be more oriented toward accelerated regulatory approval rather than comprehensive long-term outcome assessment. (**Figure 7B**, **Supplementary Figure 2**).

Geographically, most studies were conducted in the United States (n = 92), China (n = 39), and the United Kingdom (n = 37). The United States ranked highest in both the total number of studies and the proportion of positive outcomes (“YES”, 29%) (**Figure 7C**). These differences may be attributed to more advanced clinical trial infrastructure, variations in participant characteristics, or inconsistencies in reporting standards. In Singapore, although the number of studies was relatively small, the “YES” success rate was comparatively high. This may reflect the country’s centralized, high-quality academic research network and its greater reliance on industry-sponsored multicenter trials, which are typically characterized by more rigorous design and regulatory oversight.

In terms of sponsorship, biopharmaceutical companies were the dominant contributors, sponsoring 122 trials (47%) and accounting for 32 “YES” outcomes (26%). Cancer institute (n = 30, 12%, “YES”: n = 7, 23%) and University (n = 22, 9%, “YES”: n = 6, 27%) also played significant roles. In China, biopharmaceutical companies led trial activity (n = 17, 6.59%) and were responsible for all reported “YES” results (n = 3, 5.66%) (**Supplementary Tables 7, 8**).

## 4 Discussion

This study utilized bibliometric analysis and clinical trial review to assess global trends in PD-1/PD-L1 research related to RCC from 2005 to 2024. Annual publication trends revealed a slow developmental phase prior to 2012, followed by rapid acceleration. This surge coincided with the landmark study by Topalian et al., which validated PD-1/PD-L1 as immunotherapeutic targets and initiated a wave of related investigations (32). The peak observed in 2021 likely reflects a culmination of key drug approvals (e.g., Lenvatinib plus Pembrolizumab) (31), the impact of COVID-19 on scientific output and research direction, and a shift of attention toward novel targets such as CTLA-4 and LAG-3 (33).

The global landscape of PD-1/PD-L1 research reflects a dynamic interplay of scientific progress, policy direction, and collaborative networks. While the United States maintains its leadership in terms of publication impact and network centrality, the rapid rise of China since 2016 signals a growing global engagement. However, the citation-per-publication gap may reflect challenges such as limited participation in multinational trials, lower representation in high-impact journals, or differing research priorities. Beyond national comparisons, global collaborations—particularly those involving multi-center clinical trials and translational studies—have become essential in addressing complex issues such as resistance mechanisms, biomarker development, and therapeutic sequencing (34, 35). Thus, rather than focusing solely on bibliometric disparities, future efforts should prioritize fostering inclusive, high-quality international research that drives clinical innovation and improves outcomes for RCC patients worldwide.

Analysis of prolific journals and authors reveals that impactful research often emerges from large-scale clinical trials led by experts such as Choueiri TK, Motzer RJ, and Powles T. These trials—CheckMate 214 (21), CheckMate 9ER (36), and METEOR (37)- demonstrated substantial clinical value for ICIs. For example, Nivolumab combined with Ipilimumab improved OS, objective response rate (ORR), and progression-free survival (PFS), with fewer adverse events in advanced RCC with durable responses (38), as confirmed in long-term follow-up studies. The 8-year follow-up results of this trail demonstrated sustained survival benefits, durable response and a manageable safety profile, reinforcing its status as a valid first-line treatment option (38). Motzer’s team continues to investigate clinical trials and combination strategies, including Tivozanib with Nivolumab, Cabozantinib with Nivolumab and Ipilimumab, and immunotherapy regimens for non-clear cell renal cell carcinoma (39–41). Their research has revolutionized RCC treatment, contributing significantly to the development of more effective and personalized therapeutic strategies. The dual-map overlay of journals further suggests an active knowledge flow from basic immunology to translational and clinical applications, confirming the maturity and integration of this research field. One methodological consideration in interpreting co-citation results is the potential redundancy arising from overlapping author groups. In our analysis, we observed that several of the most frequently co-cited publications—including multiple landmark trials—were authored or co-authored by a small group of highly prolific investigators, notably Motzer RJ and colleagues. This concentration may introduce bias by artificially inflating network centrality and clustering metrics, particularly in a field with a relatively tight-knit research community and few pivotal trialists. To address this issue, we cross-checked author networks and co-citation clusters to identify redundancies and overlapping contributions. While we did not exclude these studies from the network (to preserve the integrity of citation-based relationships), we acknowledge that their cumulative influence may reflect both scientific impact and authorship overlap. This phenomenon underscores the importance of interpreting centrality measures in conjunction with qualitative insights—such as study design, clinical impact, and independent replication—rather than relying solely on bibliometric indicators. Future studies could adopt author-level de-duplication or fractional counting methods to more accurately estimate unique scientific contributions within co-citation networks.

Keyword clustering and co-citation burst analysis revealed a distinct chronological transition. Early research emphasized basic mechanisms (e.g., immune cell activation, PD-L1 expression). Between 2012 and 2017, keyword bursts such as “immune checkpoint” and “nivolumab” indicated clinical validation and drug development. After 2018, terms like “prognosis,” “tyrosine kinase inhibitors,” and “resistance” emerged, suggesting refinement in therapeutic strategies, combination therapies, and focus on long-term management. Keywords such as “immune-related adverse events (irAEs) “ and “tumor microenvironment” point toward increasing attention to patient safety, treatment resistance, and immunotherapy precision. In particular, the growing prominence of irAEs as a research hotspot reflects both the expanding use of ICIs and the need for better toxicity management. IrAEs range from mild dermatologic reactions to life-threatening endocrinopathies or pneumonitis, posing significant clinical challenges (42). As immunotherapy moves into earlier lines of treatment and combination regimens, managing irAEs becomes increasingly complex. Current research is focusing on predictive biomarkers for toxicity, mechanisms of immune dysregulation, and optimized treatment algorithms that balance efficacy and safety (43). However, a major challenge remains: integrating real-time toxicity data into clinical decision-making frameworks. This requires standardized irAEs reporting, long-term follow-up data, and risk-benefit models that inform personalized treatment selection (44, 45). The recent rise in “gene expression” and “whole exome sequencing” reflects a shift toward genomics-guided precision oncology, where biomarker discovery and patient stratification become central (46). Research on the tumor immune microenvironment (TME) and whole-exome sequencing (WES) has increasingly shaped the direction of RCC therapy. TME studies have identified the functional heterogeneity of immune cell populations (e.g., exhausted T cells, immunosuppressive macrophages), influencing therapeutic response and resistance to PD-1/PD-L1 blockade (47). These insights have led to strategies aiming to remodel the TME or co-target multiple immune checkpoints. Similarly, WES enables comprehensive detection of somatic mutations and neoantigen landscapes, allowing clinicians to identify high-TMB or specific mutations (e.g., PBRM1, BAP1, SETD2) predictive of ICI responsiveness (48). Integrating TME profiling with WES facilitates the development of individualized combination regimens and enhances patient stratification, ultimately improving therapeutic outcomes (49).

Our analysis of 258 clinical trials provides valuable context for understanding the translational landscape of PD-1/PD-L1 therapies in RCC. Most studies were early-phase, which may be attributed to challenges such as prolonged follow-up periods, high costs, and stringent regulatory hurdles associated with late-phase trials. Additionally, the evolving therapeutic landscape and increasing reliance on biomarker-driven patient stratification necessitate adaptive trial designs, which can further delay the initiation or completion of traditional phase 3 studies. “YES” results were concentrated in trials lasting 2–5 years, whereas long-duration studies remained scarce, possibly due to funding limitations, slow accrual, or regulatory hurdles. Interestingly, while countries like China contributed a high volume of studies, nations such as Singapore exhibited a disproportionately high rate of “YES” outcomes despite producing fewer trials. This suggests that different research strategies or funding models may influence not only the quantity but also the quality of output. For instance, sponsor analysis revealed that biopharmaceutical companies dominated in total trial numbers, yet academic institutions and cancer centers demonstrated a higher proportion of successful outcomes—potentially reflecting stricter adherence to study design and endpoint rigor. Although formal statistical comparisons (e.g., chi-square tests) were not performed on geographic heatmaps due to data limitations, these observed disparities highlight the need for future studies to incorporate robust validation methods when evaluating regional differences in research efficiency and success. These findings underscore how national research strategies and institutional priorities may shape not only the scale but also the clinical value of immunotherapy trials in RCC.

Overall, this study underscores the robust evolution of PD-1/PD-L1 research in RCC and its increasing clinical translation, as the field transitions from validation toward optimization and personalization. Based on our findings, several future research directions are suggested. These include the development of next-generation immunotherapies—such as antibody-drug conjugates (ADCs), tumor vaccines, and RNA-based agents—to overcome resistance and expand therapeutic options (50–52). The identification and validation of predictive biomarkers remain critical for improving patient stratification and guiding treatment decisions. Additionally, the use of advanced preclinical models—such as patient−derived xenografts (PDX) and organoids—will facilitate mechanistic studies and the testing of novel immunotherapy combinations (53). Future clinical trials should address current limitations by ensuring balanced trial phases, improving the representation of diverse populations, and incorporating comprehensive endpoints such as patient-reported outcomes and quality of life measures. Importantly, integrating bibliometric insights with clinical data and multi-omic platforms (genomics, transcriptomics, proteomics) will be essential for refining precision immunotherapy strategies and accelerating clinical translation in RCC.

Our study has some limitations. It only included English-language publications from the WoSCC, potentially excluding relevant studies from other databases (e.g., Scopus, PubMed, Embase) or non-English sources. Additionally, while the bibliometric analysis spans two decades, the clinical trial data only cover approximately 10 years, limiting temporal alignment between the two datasets. Moreover, citation-based metrics such as centrality and co-citation counts may be influenced by journal impact factors, publication timing, and access status, which could introduce bias in evaluating academic influence. Additionally, this study did not formally adjust for potential publication bias factors such as open-access availability, language restriction, or journal impact factor stratification. These variables may influence citation patterns and potentially skew the identification of research hotspots. As such, the bibliometric findings should be interpreted with caution, particularly when inferring scientific influence solely from citation-based metrics. Another limitation is that the clinical trial data analysis was largely descriptive and lacked comparative statistical testing across countries, study types, or funding sources. Finally, this study did not incorporate meta-analyses or real-world clinical outcomes, which are important for assessing treatment effectiveness and safety. Future work should aim to integrate bibliometric, clinical, and multi-omic data to better guide precision immunotherapy in RCC.

## 5 Conclusion

In summary, this study analyzes the research progress on PD-1/PD-L1 in RCC treatment from 2005 to 2024, integrating bibliometric indicators and clinical trial data. It objectively evaluates the contributions of countries, institutions, authors, journals, research hotspots, and emerging trends in this field. The analysis shows that PD-1/PD-L1 combined with VEGF-targeted therapies remains a central research focus, with sustained interest in immune-related adverse events, drug resistance, and prognostic outcomes. Meanwhile, research is gradually shifting toward advanced areas such as the tumor immune microenvironment, whole exome sequencing (WES), and tumor mutational burden (TMB), aiming to identify reliable predictive biomarkers. Ongoing efforts to explore novel ICIs combinations and improve biomarker-guided patient stratification will further promote personalized treatment strategies. Although most clinical trials remain in early phases and lack long-term validation, translational progress has already begun to shape the future of precision immunotherapy in RCC.

## Data Availability

The original contributions presented in the study are included in the article/**Supplementary Material**. Further inquiries can be directed to the corresponding authors.

## References

[1] Bukavina L Bensalah K Bray F Carlo M Challacombe B Karam JA . Epidemiology of renal cell carcinoma: 2022 update. Eur Urol. (2022) 82:529–42. doi: 10.1016/j.eururo.2022.08.019, PMID: 36100483

[2] Hsieh JJ Purdue MP Signoretti S Swanton C Albiges L Schmidinger M . Renal cell carcinoma. Nat Rev Dis Primers. (2017) 3:17009. doi: 10.1038/nrdp.2017.9, PMID: 28276433 PMC5936048

[3] Rose TL Kim WY . Renal cell carcinoma: A review. Jama. (2024) 332:1001–10. doi: 10.1001/jama.2024.12848, PMID: 39196544 PMC11790279

[4] Dasp J Des RS Dasdα CM Das TC Livinalli IC Bertoncelli ACZ . An overview of renal cell carcinoma hallmarks, drug resistance, and adjuvant therapies. Cancer Diagn Progn. (2023) 3:616–34. doi: 10.21873/cdp.10264, PMID: 37927802 PMC10619564

[5] Zhang Q Ren H Ge L Zhang W Song F Huang P . A review on the role of long non-coding RNA and microRNA network in clear cell renal cell carcinoma and its tumor microenvironment. Cancer Cell Int. (2023) 23:16. doi: 10.1186/s12935-023-02861-6, PMID: 36732762 PMC9893571

[6] Motzer RJ Jonasch E Agarwal N Alva A Baine M Beckermann K . Kidney cancer, version 3.2022, NCCN clinical practice guidelines in oncology. J Natl Compr Canc Netw. (2022) 20:71–90. doi: 10.6004/jnccn.2022.0001, PMID: 34991070 PMC10191161

[7] Powles T Plimack ER Soulières D Waddell T Stus V Gafanov R . Pembrolizumab plus axitinib versus sunitinib monotherapy as first-line treatment of advanced renal cell carcinoma (KEYNOTE-426): extended follow-up from a randomised, open-label, phase 3 trial. Lancet Oncol. (2020) 21:1563–73. doi: 10.1016/S1470-2045(20)30436-8, PMID: 33284113

[8] Liu Q Guan Y Li S . Programmed death receptor (PD-)1/PD-ligand (L)1 in urological cancers: the “all-around warrior” in immunotherapy. Mol Cancer. (2024) 23:183. doi: 10.1186/s12943-024-02095-8, PMID: 39223527 PMC11367915

[9] Zhang C Duan Y Xia M Dong Y Chen Y Zheng L . TFEB Mediates Immune Evasion and Resistance to mTOR Inhibition of Renal Cell Carcinoma via Induction of PD-L1. Clin Cancer Res. (2019) 25:6827–38. doi: 10.1158/1078-0432.CCR-19-0733, PMID: 31383732

[10] Moreira M Pobel C Epaillard N Simonaggio A Oudard S Vano YA . Resistance to cancer immunotherapy in metastatic renal cell carcinoma. Cancer Drug Resist. (2020) 3:454–71. doi: 10.20517/cdr.2020.16, PMID: 35582435 PMC8992500

[11] Ochoa AC Zea AH Hernandez C Rodriguez PC . Arginase, prostaglandins, and myeloid-derived suppressor cells in renal cell carcinoma. Clin Cancer Res. (2007) 13:721s–6s. doi: 10.1158/1078-0432.CCR-06-2197, PMID: 17255300

[12] Zhang J Peng Q Fan J Liu F Chen H Bi X . Single-cell and spatial transcriptomics reveal SPP1-CD44 signaling drives primary resistance to immune checkpoint inhibitors in RCC. J Transl Med. (2024) 22:1157. doi: 10.1186/s12967-024-06018-5, PMID: 39736762 PMC11687132

[13] Shapiro DD Dolan B Laklouk IA Rassi S Lozar T Emamekhoo H . Understanding the tumor immune microenvironment in renal cell carcinoma. Cancers (Basel). (2023) 15(9):2500. doi: 10.3390/cancers15092500, PMID: 37173966 PMC10177515

[14] Mustafa M Ahmad R Tantry IQ Ahmad W Siddiqui S Alam M . Apoptosis: A comprehensive overview of signaling pathways, morphological changes, and physiological significance and therapeutic implications. Cells. (2024) 13(22):1838. doi: 10.3390/cells13221838, PMID: 39594587 PMC11592877

[15] Lasorsa F di Meo NA Rutigliano M Milella M Ferro M Pandolfo SD . Immune checkpoint inhibitors in renal cell carcinoma: molecular basis and rationale for their use in clinical practice. Biomedicines. (2023) 11(4):1071. doi: 10.3390/biomedicines11041071, PMID: 37189689 PMC10136190

[16] Ohaegbulam KC Assal A Lazar-Molnar E Yao Y Zang X . Human cancer immunotherapy with antibodies to the PD-1 and PD-L1 pathway. Trends Mol Med. (2015) 21:24–33. doi: 10.1016/j.molmed.2014.10.009, PMID: 25440090 PMC4282825

[17] Sun C Mezzadra R Schumacher TN . Regulation and function of the PD-L1 checkpoint. Immunity. (2018) 48:434–52. doi: 10.1016/j.immuni.2018.03.014, PMID: 29562194 PMC7116507

[18] Thompson RH Dong H Lohse CM Leibovich BC Blute ML Cheville JC . PD-1 is expressed by tumor-infiltrating immune cells and is associated with poor outcome for patients with renal cell carcinoma. Clin Cancer Res. (2007) 13:1757–61. doi: 10.1158/1078-0432.CCR-06-2599, PMID: 17363529

[19] Pardoll DM . The blockade of immune checkpoints in cancer immunotherapy. Nat Rev Cancer. (2012) 12:252–64. doi: 10.1038/nrc3239, PMID: 22437870 PMC4856023

[20] Motzer RJ Escudier B McDermott DF George S Hammers HJ Srinivas S . Nivolumab versus everolimus in advanced renal-cell carcinoma. N Engl J Med. (2015) 373:1803–13. doi: 10.1056/NEJMoa1510665, PMID: 26406148 PMC5719487

[21] Motzer RJ Tannir NM McDermott DF Arén Frontera O Melichar B Choueiri TK . Nivolumab plus Ipilimumab versus Sunitinib in Advanced Renal-Cell Carcinoma. N Engl J Med. (2018) 378:1277–90. doi: 10.1056/NEJMoa1712126, PMID: 29562145 PMC5972549

[22] Rini BI Plimack ER Stus V Gafanov R Hawkins R Nosov D . Pembrolizumab plus Axitinib versus Sunitinib for Advanced Renal-Cell Carcinoma. N Engl J Med. (2019) 380:1116–27. doi: 10.1056/NEJMoa1816714, PMID: 30779529

[23] Bahadoram S Davoodi M Hassanzadeh S Bahadoram M Barahman M Mafakher L . Renal cell carcinoma: an overview of the epidemiology, diagnosis, and treatment. G Ital Nefrol. (2022) 39(3):2022-vol3., PMID: 35819037

[24] Khan Y Slattery TD Pickering LM . Individualizing systemic therapies in first line treatment and beyond for advanced renal cell carcinoma. Cancers (Basel). (2020) 12(12):3750. doi: 10.3390/cancers12123750, PMID: 33322163 PMC7764621

[25] Ma X Chen J Chen S Lan X Wei Z Gao H . Immunotherapy for renal cell carcinoma: New therapeutic combinations and adverse event management strategies: A review. Med (Baltimore). (2024) 103:e38991. doi: 10.1097/MD.0000000000038991, PMID: 39058879 PMC11272340

[26] Tenold M Ravi P Kumar M Bowman A Hammers H Choueiri TK . Current approaches to the treatment of advanced or metastatic renal cell carcinoma. Am Soc Clin Oncol Educ Book. (2020) 40:1–10. doi: 10.1200/EDBK_279881, PMID: 32239988

[27] van Eck NJ Waltman L . Software survey: VOSviewer, a computer program for bibliometric mapping. Scientometrics. (2010) 84:523–38. doi: 10.1007/s11192-009-0146-3, PMID: 20585380 PMC2883932

[28] Synnestvedt MB Chen C Holmes JH . CiteSpace II: visualization and knowledge discovery in bibliographic databases. AMIA Annu Symp Proc. (2005) 2005:724–8., PMID: 16779135 PMC1560567

[29] Motzer RJ Penkov K Haanen J Rini B Albiges L Campbell MT . Avelumab plus Axitinib versus Sunitinib for Advanced Renal-Cell Carcinoma. N Engl J Med. (2019) 380:1103–15. doi: 10.1056/NEJMoa1816047, PMID: 30779531 PMC6716603

[30] Motzer RJ Rini BI McDermott DF Redman BG Kuzel TM Harrison MR . Nivolumab for metastatic renal cell carcinoma: results of a randomized phase II trial. J Clin Oncol. (2015) 33:1430–7. doi: 10.1200/JCO.2014.59.0703, PMID: 25452452 PMC4806782

[31] Motzer R Alekseev B Rha SY Porta C Eto M Powles T . Lenvatinib plus pembrolizumab or everolimus for advanced renal cell carcinoma. N Engl J Med. (2021) 384:1289–300. doi: 10.1056/NEJMoa2035716, PMID: 33616314

[32] Topalian SL Hodi FS Brahmer JR Gettinger SN Smith DC McDermott DF . Safety, activity, and immune correlates of anti-PD-1 antibody in cancer. N Engl J Med. (2012) 366:2443–54. doi: 10.1056/NEJMoa1200690, PMID: 22658127 PMC3544539

[33] Li YQ Chen XM Si GF Yuan XM . Progress of lymphocyte activation gene 3 and programmed cell death protein 1 antibodies for cancer treatment: A review. Biomol Biomed. (2024) 24:764–74. doi: 10.17305/bb.2024.10339, PMID: 38581716 PMC11293232

[34] Motzer RJ Porta C Eto M Hutson TE Rha SY Merchan JR . Biomarker analyses from the phase III randomized CLEAR trial: lenvatinib plus pembrolizumab versus sunitinib in advanced renal cell carcinoma. Ann Oncol. (2025) 36:375–86. doi: 10.1016/j.annonc.2024.12.003, PMID: 39672382 PMC12459081

[35] Pal SK Albiges L Tomczak P Suárez C Voss MH de Velasco G . Atezolizumab plus cabozantinib versus cabozantinib monotherapy for patients with renal cell carcinoma after progression with previous immune checkpoint inhibitor treatment (CONTACT-03): a multicentre, randomised, open-label, phase 3 trial. Lancet. (2023) 402:185–95. doi: 10.1016/S0140-6736(23)00922-4, PMID: 37290461 PMC11017728

[36] Choueiri TK Powles T Burotto M Escudier B Bourlon MT Zurawski B . Nivolumab plus Cabozantinib versus Sunitinib for Advanced Renal-Cell Carcinoma. N Engl J Med. (2021) 384:829–41. doi: 10.1056/NEJMoa2026982, PMID: 33657295 PMC8436591

[37] Choueiri TK Escudier B Powles T Mainwaring PN Rini BI Donskov F . Cabozantinib versus everolimus in advanced renal-cell carcinoma. N Engl J Med. (2015) 373:1814–23. doi: 10.1056/NEJMoa1510016, PMID: 26406150 PMC5024539

[38] Tannir NM Albigès L McDermott DF Burotto M Choueiri TK Hammers HJ . Nivolumab plus ipilimumab versus sunitinib for first-line treatment of advanced renal cell carcinoma: extended 8-year follow-up results of efficacy and safety from the phase III CheckMate 214 trial. Ann Oncol. (2024) 35:1026–38. doi: 10.1016/j.annonc.2024.07.727, PMID: 39098455 PMC11907766

[39] Choueiri TK Albiges L Barthélémy P Iacovelli R Emambux S Molina-Cerrillo J . Tivozanib plus nivolumab versus tivozanib monotherapy in patients with renal cell carcinoma following an immune checkpoint inhibitor: results of the phase 3 TiNivo-2 Study. Lancet. (2024) 404:1309–20. doi: 10.1016/S0140-6736(24)01758-6, PMID: 39284329 PMC12208211

[40] Go C Okumura H Miura Y . Cabozantinib plus nivolumab and ipilimumab in renal-cell carcinoma. N Engl J Med. (2023) 389:477. doi: 10.1056/NEJMc2306786, PMID: 37530835

[41] Fitzgerald KN Lee CH Voss MH Carlo MI Knezevic A Peralta L . Cabozantinib plus nivolumab in patients with non-clear cell renal cell carcinoma: updated results from a phase 2 trial. Eur Urol. (2024) 86:90–4. doi: 10.1016/j.eururo.2024.04.025, PMID: 38782695 PMC11970537

[42] Martins F Sofiya L Sykiotis GP Lamine F Maillard M Fraga M . Adverse effects of immune-checkpoint inhibitors: epidemiology, management and surveillance. Nat Rev Clin Oncol. (2019) 16:563–80. doi: 10.1038/s41571-019-0218-0, PMID: 31092901

[43] Les I Martínez M Pérez-Francisco I Cabero M Teijeira L Arrazubi V . Predictive biomarkers for checkpoint inhibitor immune-related adverse events. Cancers (Basel). (2023) 15(5):1629. doi: 10.3390/cancers15051629, PMID: 36900420 PMC10000735

[44] Chen TW Razak AR Bedard PL Siu LL Hansen AR . A systematic review of immune-related adverse event reporting in clinical trials of immune checkpoint inhibitors. Ann Oncol. (2015) 26:1824–9. doi: 10.1093/annonc/mdv182, PMID: 25888611

[45] Naidoo J Murphy C Atkins MB Brahmer JR Champiat S Feltquate D . Society for Immunotherapy of Cancer (SITC) consensus definitions for immune checkpoint inhibitor-associated immune-related adverse events (irAEs) terminology. J Immunother Cancer. (2023) 11(3):e006398. doi: 10.1136/jitc-2022-006398, PMID: 37001909 PMC10069596

[46] Fay AP de Velasco G Ho TH Van Allen EM Murray B Albiges L . Whole-exome sequencing in two extreme phenotypes of response to VEGF-targeted therapies in patients with metastatic clear cell renal cell carcinoma. J Natl Compr Canc Netw. (2016) 14:820–4. doi: 10.6004/jnccn.2016.0086, PMID: 27407122 PMC5582541

[47] Wang Y Wang H Yao H Li C Fang JY Xu J . Regulation of PD-L1: emerging routes for targeting tumor immune evasion. Front Pharmacol. (2018) 9:536. doi: 10.3389/fphar.2018.00536, PMID: 29910728 PMC5992436

[48] Braun DA Street K Burke KP Cookmeyer DL Denize T Pedersen CB . Progressive immune dysfunction with advancing disease stage in renal cell carcinoma. Cancer Cell. (2021) 39:632–48.e8. doi: 10.1016/j.ccell.2021.02.013, PMID: 33711273 PMC8138872

[49] Krieg C Nowicka M Guglietta S Schindler S Hartmann FJ Weber LM . High-dimensional single-cell analysis predicts response to anti-PD-1 immunotherapy. Nat Med. (2018) 24:144–53. doi: 10.1038/nm.4466, PMID: 29309059

[50] Sganga S Riondino S Iannantuono GM Rosenfeld R Roselli M Torino F . Antibody-drug conjugates for the treatment of renal cancer: A scoping review on current evidence and clinical perspectives. J Pers Med. (2023) 13(9):1339. doi: 10.3390/jpm13091339, PMID: 37763107 PMC10532725

[51] Wei YC Sticca RP Li J Holmes LM Burgin KE Jakubchak S . Combined treatment of dendritoma vaccine and low-dose interleukin-2 in stage IV renal cell carcinoma patients induced clinical response: A pilot study. Oncol Rep. (2007) 18:665–71. doi: 10.3892/or.18.3.665, PMID: 17671717

[52] Deeks ED . Belzutifan: first approval. Drugs. (2021) 81:1921–7. doi: 10.1007/s40265-021-01606-x, PMID: 34613603

[53] Chen Q Sun X Li Y Yang X Yang X Xu H . The potential of organoids in renal cell carcinoma research. BMC Urol. (2024) 24:120. doi: 10.1186/s12894-024-01511-x, PMID: 38858665 PMC11165752

