# Supplementary material for: Integrating clinical trial landscapes and bibliometric analysis: unveiling the impact of PD-1/PD-L1 inhibitors on renal cell carcinoma research and therapeutic trajectories summary
Source: Front Immunol. 2025 Jul 23;16:1578838. doi: 10.3389/fimmu.2025.1578838 (PMC12325349; doi:10.3389/fimmu.2025.1578838)
Supplement: Supplementary file 1 [file DataSheet1.docx]

Supplementary Material

**Supplementary Table 1**: Targeted Therapy and Immunotherapy Agents for Renal Cancer Mechanisms of Action and Classification.

| **Drug Name(s)** | **Mechanism of Action** | **Classification** |
| --- | --- | --- |
| Sunitinib | VEGF pathway inhibition | TKI |
| Axitinib | VEGF pathway inhibition | TKI |
| Cabozantinib | VEGF/MET/AXL inhibition | TKI |
| Tivozanib | VEGF pathway inhibition | TKI |
| Nivolumab | PD-1 inhibition | ICI |
| Pembrolizumab | PD-1 inhibition | ICI |
| Ipilimumab | CTLA-4 inhibition | ICI |
| Nivolumab + Ipilimumab | Dual checkpoint blockade (PD-1 + CTLA-4) | ICI Combination |
| Nivolumab + Cabozantinib | Checkpoint + TKI combo (PD-1 + VEGFR) | Mixed (ICI + TKI) |
| Tivozanib + Nivolumab | Checkpoint + TKI combo (PD-1 + VEGFR) | Mixed (ICI + TKI) |

**Supplementary Table 2**: Table of institutional published literature.

| **Rank** | **Institution** | **Country** | **Number of studies** | **Total citations** | **Average citation** |
| --- | --- | --- | --- | --- | --- |
| 1 | Harvard University | USA | 157 | 31947 | 203.48 |
| 2 | Dana-Farber Cancer Institute | France | 118 | 28697 | 243.19 |
| 3 | Unicancer | USA | 94 | 16390 | 174.36 |
| 4 | University of Texas System | USA | 93 | 11096 | 119.31 |
| 5 | Harvard Medical School | USA | 79 | 9857 | 124.77 |
| 6 | Memorial Sloan Kettering Cancer Center | USA | 77 | 25663 | 333.29 |
| 7 | Beth Israel Deaconess Medical Center | USA | 71 | 22532 | 317.35 |
| 8 | UTMD Anderson Cancer Center | USA | 64 | 16404 | 256.31 |
| 9 | Gustave Roussy | France | 63 | 12723 | 201.95 |
| 10 | Brigham & Women's Hospital | USA | 62 | 12165 | 196.21 |

**Supplementary Table 3**: Table of journal publications.

| **Rank** | **Journal** | **Article counts** | **Percentage (1597)** | **IF (2023)** | **Quartile in category** |
| --- | --- | --- | --- | --- | --- |
| 1 | JOURNAL FOR IMMUNOTHERAPY OF CANCER | 66 | 4.13% | 10.3 | Q1 |
| 2 | FRONTIERS IN ONCOLOGY | 63 | 3.94% | 3.5 | Q3 |
| 3 | CANCERS | 48 | 3.01% | 4.5 | Q2 |
| 4 | FRONTIERS IN IMMUNOLOGY | 41 | 2.57% | 5.7 | Q2 |
| 5 | CLINICAL CANCER RESEARCH | 39 | 2.44% | 10 | Q1 |
| 6 | ONCOIMMUNOLOGY | 31 | 1.94% | 6.5 | Q2 |
| 7 | CLINICAL GENITOURINARY CANCER | 27 | 1.69% | 2.3 | Q3 |
| 8 | CANCER IMMUNOLOGY IMMUNOTHERAPY | 26 | 1.63% | 4.6 | Q2 |
| 9 | INTERNATIONAL JOURNAL OF MOLECULAR SCIENCES | 25 | 1.57% | 4.9 | Q2 |
| 10 | CANCER IMMUNOLOGY RESEARCH | 24 | 1.50% | 8.1 | Q1 |

**Supplementary Table 4**: Co-citation table of journals.

| **Rank** | **Cited Journal** | **Co-Citation** | **IF (2023)** | **Quartile in category** |
| --- | --- | --- | --- | --- |
| 1 | NEW ENGLAND JOURNAL OF MEDICINE | 1300 | 96.2 | Q1 |
| 2 | JOURNAL OF CLINICAL ONCOLOGY | 1114 | 42.1 | Q1 |
| 3 | CLINICAL CANCER RESEARCH | 1085 | 10 | Q1 |
| 4 | LANCET ONCOLOGY | 829 | 41.6 | Q1 |
| 5 | CANCER RESEARCH | 806 | 12.5 | Q1 |
| 6 | ANNALS OF ONCOLOGY | 791 | 56.7 | Q1 |
| 7 | NATURE | 737 | 50.5 | Q1 |
| 8 | NATURE MEDICINE | 671 | 58.7 | Q1 |
| 9 | LANCET | 633 | 98.4 | Q1 |
| 10 | JOURNAL FOR IMMUNOTHERAPY OF CANCER | 631 | 10.3 | Q1 |

**Supplementary Table 5**: Author's publications and co-citations table.

| **Rank** | **Author** | **Count** | **Rank** | **Co-cited author** | **Citation** |
| --- | --- | --- | --- | --- | --- |
| 1 | Choueiri TK | 56 | 1 | Motzer RJ | 951 |
| 2 | Mcdermott DF | 44 | 2 | Choueiri TK | 554 |
| 3 | Motzer RJ | 31 | 3 | Rini BI | 543 |
| 4 | Powles T | 31 | 4 | Mcdermott DF | 408 |
| 5 | Atkins MB | 29 | 5 | Topalian SL | 399 |
| 6 | Albiges L | 28 | 6 | Escudier B | 299 |
| 7 | Escudier B | 26 | 7 | Brahmer JR | 290 |
| 8 | Massari F | 21 | 8 | Thompson RH | 285 |
| 9 | Bedke J | 20 | 9 | Powles T | 281 |
| 10 | Signoretti S | 20 | 10 | Hodi FS | 223 |

**Supplementary Table 6**: Co-citation table of literature.

| **Rank** | **Title** | **Journal** | **author(s)** | **Total citations** |
| --- | --- | --- | --- | --- |
| 1 | Nivolumab plus Ipilimumab versus Sunitinib in Advanced Renal-Cell Carcinoma | NEW ENGLAND JOURNAL OF MEDICINE | Motzer RJ | 393 |
| 2 | Nivolumab versus Everolimus in Advanced Renal-Cell Carcinoma | NEW ENGLAND JOURNAL OF MEDICINE | Motzer RJ | 357 |
| 3 | Pembrolizumab plus Axitinib versus Sunitinib for Advanced Renal-Cell Carcinoma | NEW ENGLAND JOURNAL OF MEDICINE | Rini BI | 324 |
| 4 | Avelumab plus Axitinib versus Sunitinib for Advanced Renal-Cell Carcinoma | NEW ENGLAND JOURNAL OF MEDICINE | Motzer RJ | 275 |
| 5 | Safety, Activity, and Immune Correlates of Anti-PD-1 Antibody in Cancer | NEW ENGLAND JOURNAL OF MEDICINE | Topalian SL | 172 |
| 6 | Clinical activity and molecular correlates of response to atezolizumab alone or in combination with bevacizumab versus sunitinib in renal cell carcinoma | NATURE MEDICINE | McDermott DF | 152 |
| 7 | Atezolizumab plus bevacizumab versus sunitinib in patients with previously untreated metastatic renal cell carcinoma (IMmotion151): a multicentre, open-label, phase 3, randomised controlled trial | LANCET | Rini BI | 146 |
| 8 | Nivolumab for Metastatic Renal Cell Carcinoma: Results of a Randomized Phase II Trial | JOURNAL OF CLINICAL ONCOLOGY | Motzer RJ | 126 |
| 9 | Nivolumab plus Cabozantinib versus Sunitinib for Advanced Renal-Cell Carcinoma | NEW ENGLAND JOURNAL OF MEDICINE | Choueiri TK | 123 |
| 10 | Lenvatinib plus Pembrolizumab or Everolimus for Advanced Renal Cell Carcinoma | NEW ENGLAND JOURNAL OF MEDICINE | Motzer R | 117 |

**Supplementary Table 7**: Studies count by sponsor type.

| **Sponsor Type** | **Total Studies** | **Yes Studies** | **Total Studies Percentage** | **Yes Studies Percentage** |
| --- | --- | --- | --- | --- |
| Biopharmaceutical company | 122 | 32 | 47% | 26% |
| Hospitals | 37 | 1 | 14% | 3% |
| Cancer institute | 30 | 7 | 12% | 23% |
| Biotech company | 26 | 1 | 10% | 4% |
| University | 22 | 6 | 9% | 27% |
| Individuals | 20 | 6 | 8% | 30% |
| Medical equipment company | 1 | 0 | 0% | 0% |
| Total | 258 | 53 | 100% | 21% |

**Supplementary Table 8**: Studies count by sponsor type of China.

| **Sponsor Type** | **Total Studies** | **Yes Studies** | **Total Studies Percentage** | **Yes Studies Percentage** |
| --- | --- | --- | --- | --- |
| Biopharmaceutical company | 17 | 3 | 6.59% | 5.66% |
| Hospitals | 13 | 0 | 5.04% | 0.00% |
| Biotech company | 2 | 0 | 1.55% | 0.00% |
| University | 2 | 0 | 0.78% | 0.00% |
| Individuals | 4 | 0 | 0.78% | 0.00% |
| Medical equipment company | 1 | 0 | 0.39% | 0.00% |
| Total | 39 | 3 | 15% | 5.66% |


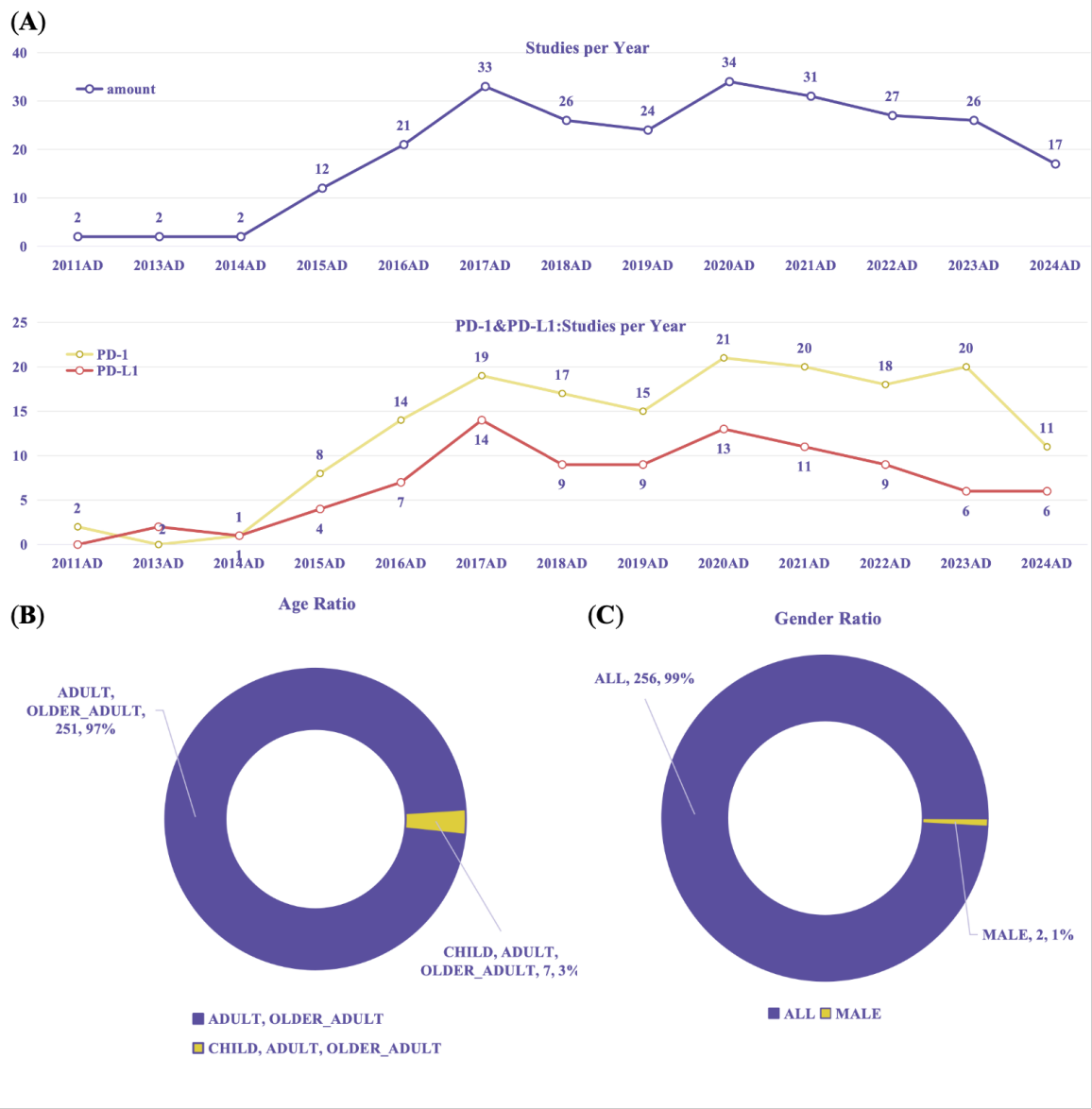


**Supplementary Figure 1**: (A) Number of studies per year; (B) Age ratio of the study; (C) Gender ratio of the study.


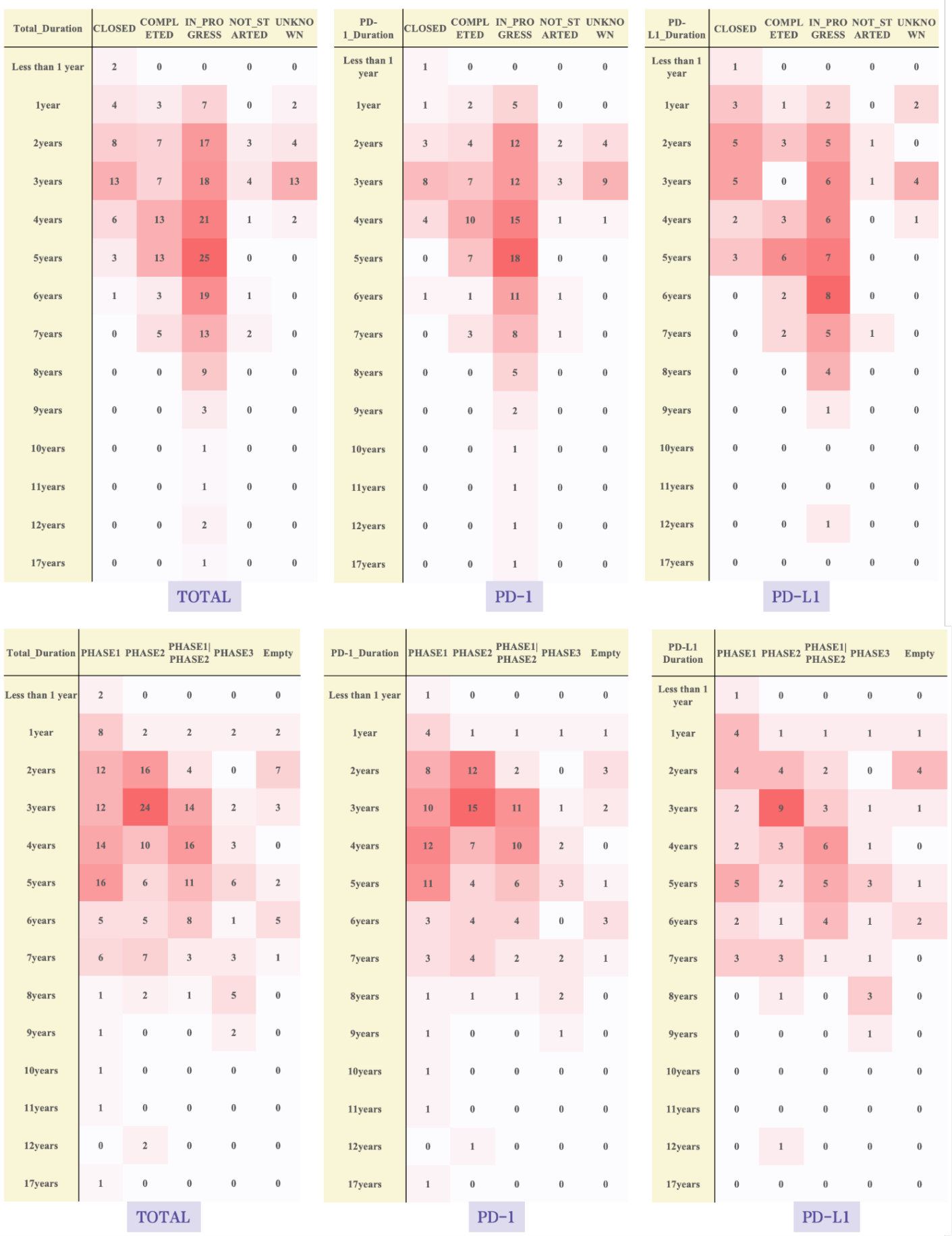


**Supplementary Figure 2**: The duration corresponds to the heat maps of the study state (top) and phase (bottom).
